# High levels of dietary stearate promote adiposity and deteriorate hepatic insulin sensitivity

**DOI:** 10.1186/1743-7075-7-24

**Published:** 2010-03-27

**Authors:** Sjoerd AA van den Berg, Bruno Guigas, Silvia Bijland, Margriet Ouwens, Peter J Voshol, Rune R Frants, Louis M Havekes, Johannes A Romijn, Ko Willems van Dijk

**Affiliations:** 1 Department of Human Genetics, Leiden University Medical Center, Leiden, The Netherlands; 2 Department of Molecular Cell Biology, Leiden University Medical Center, Leiden, The Netherlands; 3 Department of Endocrinology and Metabolic Diseases, Leiden University Medical Center, Leiden, The Netherlands; 4 Department of General Internal Medicine, Leiden University Medical Center, Leiden, The Netherlands; 5 The Netherlands Organization for Applied Scientific Research - BioSciences, Gaubius Laboratory, Leiden, The Netherlands; 6 Department of Cardiology, Leiden University Medical Center, Leiden, The Netherlands

## Abstract

**Background:**

Relatively little is known about the role of specific saturated fatty acids in the development of high fat diet induced obesity and insulin resistance. Here, we have studied the effect of stearate in high fat diets (45% energy as fat) on whole body energy metabolism and tissue specific insulin sensitivity.

**Methods:**

C57Bl/6 mice were fed a low stearate diet based on palm oil or one of two stearate rich diets, one diet based on lard and one diet based on palm oil supplemented with tristearin (to the stearate level of the lard based diet), for a period of 5 weeks. *Ad libitum *fed Oxidative metabolism was assessed by indirect calorimetry at week 5. Changes in body mass and composition was assessed by DEXA scan analysis. Tissue specific insulin sensitivity was assessed by hyperinsulinemic-euglycemic clamp analysis and Western blot at the end of week 5.

**Results:**

Indirect calorimetry analysis revealed that high levels of dietary stearate resulted in lower caloric energy expenditure characterized by lower oxidation of fatty acids. In agreement with this metabolic phenotype, mice on the stearate rich diets gained more adipose tissue mass. Whole body and tissue specific insulin sensitivity was assessed by hyperinsulinemic-euglycemic clamp and analysis of insulin induced PKB^ser473 ^phosphorylation. Whole body insulin sensitivity was decreased by all high fat diets. However, while insulin-stimulated glucose uptake by peripheral tissues was impaired by all high fat diets, hepatic insulin sensitivity was affected only by the stearate rich diets. This tissue-specific pattern of reduced insulin sensitivity was confirmed by similar impairment in insulin-induced phosphorylation of PKB^ser473 ^in both liver and skeletal muscle.

**Conclusion:**

In C57Bl/6 mice, 5 weeks of a high fat diet rich in stearate induces a metabolic state favoring low oxidative metabolism, increased adiposity and whole body insulin resistance characterized by severe hepatic insulin resistance. These results indicate that dietary fatty acid composition *per sé *rather than dietary fat content determines insulin sensitivity in liver of high fat fed C57Bl/6 mice.

## Introduction

High fat diets are widely used to study the development of obesity and insulin resistance in rodent models. The fat used in these diets often derives from natural sources, for example lard, tallow, palm oil or coca butter which contain fatty acids varying in chain-length and saturation level. Although different high fat diets clearly induce different effects [1-3], it has been a challenge to assign specific effects to individual fatty acids. This is specifically of interest and relevance for differential effects of the saturated long chain fatty acids (FA) palmitic acid (C16:0) and stearic acid (C18:0), which represent the most common nutritional long chain fatty acids [4].

Differences in dietary FA composition are of physiological relevance, since the metabolic fate of FA is dependent on chain length as well as the degree of saturation. For example, oxidative efficiency of FA decreases with increasing chain length and saturation level. In rats, after oral administration of labeled FA, the efficiency of the oxidation of saturated FA has been demonstrated to be lauric acid (C12:0) > myristic acid (C14:0) > palmitic acid (C16:0) > stearic acid (C18:0) [5]. Similar results have been found in a human study where the oxidation rate of stearic acid after a bolus administration was found to be poor in comparison to lauric acid (13% versus 41% oxidized within 9 hours after administration) [6]. Accordingly, at the cellular level, stearic acid has been described to be poorly oxidized by hepatocytes [7]. In addition to their low oxidative efficiency, saturated long chain FA are known to affect insulin sensitivity directly in a chain length dependent manner via a TLR4 dependent pathway [8-10].

In this study, we investigated whether the level of dietary stearate in high fat diets determines whole-body energy metabolism and tissue-specific insulin sensitivity. For this purpose, mice were fed for 5 weeks either a diet low in stearate or two diets naturally or artificially enriched in stearate. Whole-body metabolism was assessed by indirect calorimetry and body composition analyzed by Dual Energy X-ray Absorptiometry (DEXA). Tissue-specific insulin sensitivity was evaluated by both hyperinsulinemic-euglycemic clamp and phosphorylation of key proteins involved in the insulin signaling pathway.

## Methods

### Animals, diets and housing

All animal experiments were approved by the Animal Ethic Committee from the Leiden University Medical Center (Leiden, the Netherlands) in accordance with the principles and guidelines established by the European Convention for the Protection of Laboratory Animals.

Male C57Bl/6J mice were obtained from Charles River Laboratories at an age of 8 weeks and acclimatized up to an age of 12 weeks at the Leiden University Medical Center animal facility. Animals were housed in a controlled environment (23°C, 55% humidity) under a 12 h light-dark cycle (07:00-19:00). Food and tap water was available *ad libitum *during the whole experiment. After acclimatization mice were switched to a low-fat run-in diet (10% energy in the form of fat, D12450B, Research Diet Services, Wijk bij Duurstede, The Netherlands) for a period of 2 weeks, with fat sources consisting of palm oil (LFW) or lard (LFL) matching the later experimental high and low stearate high fat diets. The composition of the low fat run in diets was similar to the high fat diets in all respects apart from total fat content. At the age of 14 weeks, animals were randomized for body mass and switched to the high fat experimental diets (24% w/w protein, 41% w/w carbohydrate and 24% w/w fat with 45% energy in the form of fat, D12451, Research Diet Services, Wijk bij Duurstede, The Netherlands) for a period of 5 weeks. High fat diets are referred to as HFP (palm oil, 4.4% of total fat content is stearate), HFL (lard based diet, 15.0% of total fat is stearate) and HFPS (palm oil + tristearin supplementation, 13.9% of total fat is stearate) in this paper (table 1). After tristearin supplementation, stearate content of the HFPS high fat diet was comparable to the lard based high fat diet, whereas the dilution of the other fatty acids was kept to a minimum (2.3%) to avoid secondary effects due to depletion of other fatty acids (table 2).

**Table 1:** Composition of HFP, HFL, HFPS high fat diets.

Diet composition			
**Ingredient**	**grams**		

Casein, 80 Mesh	200	200	200

L-Cystine	3	3	3

Corn Starch	72.8	72.8	72.8

Maltodextrin 10	100	100	100

Sucrose	172.8	172.8	172.8

Cellulose, BW200	50	50	50

Soybean Oil	25	25	25

Palm oil	177.5	0	177.5

Tristearin	0	0	27.5

Lard	0	177.5	0

Mineral Mix S10026	10	10	10

DiCalcium Phosphate	13	13	13

Calcium Carbonate	5.5	5.5	5.5

Potassium Citrate, 1 H2O	16.5	16.5	16.5

Vitamin Mix V10001	10	10	10

Choline Bitartrate	2	2	2

FD&C Red Dye #40	0.05	0.05	0.05

total	858.15	858.15	885.65

**Table 2:** Fatty acid composition of HFP, HFL, HFPS. FA levels are represented as percentage of total fat content.

Fatty acid composition (% of total):			
	**HFP**	**HFL**	**HFPS**

C12:0	0.3	-	0.3

C14:0	0.9	0.8	0.8

C16:0	35.6	29.2	32.0

C16:1w7	0.2	2.8	0.2

C18:0	4.4	15.0	13.9

C18:1w9	40.6	43.3	36.6

C18:2w6	16.5	8.9	14.8

C18:3w3	0.7	-	0.6

C20:0	0.5	-	0.5

C20:1w9	0.1	-	0.1

C22:0	0.2	-	0.2

Total %	100	100	100

### Indirect calorimetry

Groups of 8 mice per high fat diet were subjected to individual indirect calorimetry measurements for a period of 4 consecutive days (Comprehensive Laboratory Animal Monitoring System, Columbus Instruments, Columbus Ohio, US). A period of 24 hours prior to the start of the experiment allowed the acclimatization of the animals to the cages and the single housing. Experimental analysis started at 09:00 h and continued for 72 hours. Analyzed parameters included real time food and water intake, as well as meal size, frequency and duration. Oxygen consumption (VO2) and carbon dioxide production rates (VCO2) were measured at intervals of 7 minutes. Respiratory exchange ratio (RER) as a measure for metabolic substrate choice was calculated using the following formula:

Carbohydrate and fat oxidation rates were calculated from VO2 and VCO2 using the following formulas [11]:

VO2 and VCO2 values are in mL/h. Total energy expenditure was calculated from the sum of carbohydrate and fat oxidation. Activity was monitored as infrared beam breaks in both X and Y axis.

### Body anthropometry and Dual Energy X-ray Absorptiometry (DEXA) scan analysis

Animals were subjected to DEXA scan analysis in fed conditions to avoid weight loss induced by overnight fasting. Animals were weighed and sedated by a single intra peritoneal injection of a mixture of Acepromazin (0.5 mg/kg), Midazolam (0.25 mg/kg) and Fentanyl (0.025 mg/kg). Sedated animals were scanned *in toto *using a small animal DEXA scanner (pDEXA, Norland Stratec Medizintechinik GmbH, Birkenfeld, Germany) and data were analyzed by the software supplied by the manufacturer. Fat mass and lean body mass were determined.

### Hyperinsulinemic euglycemic clamp experiments

Hyperinsulinemic - euglycemic clamp experiments were performed as described before [12-16] with minor modifications to fit our specific model. Per group 5-8 mice were clamped. Clamp experiments were performed after an overnight fast. Animals were anesthetized by ip injection with a combination of Acepromazin (0.5 mg/kg, Sanofi Santé Nutrition Animale, Libourne Cedex, France), Midazolam (0.25 mg/kg, Roche, Mijdrecht, The Netherlands) and Fentanyl (0.025 mg/kg, Janssen-Cilag, Tilburg, The Netherlands). An infusion needle was placed into a tail vein. After 60 min infusion of D-[1-14C]glucose at a rate of 0.8 μCi/h (specific activity, 9.6 GBq/mmol; Amersham, Little Chalfont, UK) to achieve steady-state levels and basal parameters were determined with 10-min intervals. Thereafter, a bolus of insulin (4.5 mU, Actrapid; Novo Nordisk, Chartres, France) was administered and the hyperinsulinemic clamp was started. Insulin was infused at a constant rate of 3.5 mU/kg.min, and D-[1-14C]glucose was infused at a rate of 0.8 μCi/h. A variable infusion of 12.5% D-glucose (in PBS) was also started to maintain euglycemic basal blood glucose levels. Blood glucose was measured with an AccuCheck hand glucose measurer (AccuCheck, Roche Diagnostics, Metronic Medical Systems, Vianen, The Netherlands) every 10 min to monitor glucose levels and adjust the glucose pump. After reaching steady state, blood samples were taken at 10-min intervals during 30 min to determine steady-state levels of [14C]glucose. An average clamp experiment took approximately 2.5 h, and anesthesia as well as body temperature was maintained throughout the procedure. For time courses of the plasma glucose levels and glucose infusion rates during the clamp are shown (see additional file 1).

### Analysis of clamp samples

Plasma insulin concentrations were measured by ELISA (Mercodia, Sweden). To measure plasma [14C]glucose activity, trichloroacetic acid (final concentration 2%) was added to 7.5 μl plasma to precipitate proteins using centrifugation. The supernatant was dried to remove water and resuspended in milliQ. The samples were counted using scintillation counting (Packard Instruments, Dowers Grove, IL).

### Calculations

The glucose turnover rate (μmol/min·kg) was calculated during the basal period and under steady-state clamp conditions as the rate of tracer infusion (dpm/min) divided by the plasma specific activity of [14C]glucose (dpm/μmol). The ratio was corrected for lean body mass. The hyperinsulinemic hepatic glucose production (HGP) was calculated as the difference between the tracer-derived rate of glucose appearance and the glucose infusion rate.

### Insulin signalling experiments

Insulin signalling analysis was performed as described before [17,18], with minor modifications to fit our specific model. Experiments were performed after overnight fast, to mimic the physiological situation of the clamp experiments. A total of 15 to 20 mice per intervention group were used in this experiment. Each group was divided in a control (PBS infusion) group and an insulin group. All animals were sedated using a mixture of Acepromazin (0.5 mg/kg), Midazolam (0.25 mg/kg) and Fentanyl (0.025 mg/kg). Custom made intravenous occlusion canulas (27G * 3/4, BD Microlance) were inserted in the tail vein and kept open by infusion of PBS. PBS or insulin (0.25 mU/min) was administered for a period of 15 minutes during the experiment. Co-infusion with glucose (1.73 umol/min) was performed in concert with insulin to maintain euglycemia. After the infusion animals were sacrificed by cervical dislocation and liver and calf muscle were harvested and snap frozen in liquid nitrogen as fast as possible. Insulin stimulated phosphorylation of PKB at serine 473 was measured and corrected for total PKB.

### Western Blot analysis

Tissues were homogenized by Ultraturax in a 10:1 (v/w) ratio of ice-cold buffer containing: 30 mM Tris.HCl (pH 7.5), 150 mM NaCl, 0.5% Triton X-100, 0.5% sodium deoxycholate, 1% SDS, 1 mM Na3VO4, 10 mM NaF and protease inhibitors cocktail (*Complete *Protease Inhibitor Cocktail, Roche Nederland BV, Woeren, The Netherlands). Homogenates were cleared by centrifugation (13.200 rpm; 15 min, 4°C) and the protein content of the supernatant was determined using a BCA protein assay kit (Thermo Fisher Scientific Inc, Rockford, IL, USA) Samples were prepared in 2 × Laemmli buffer containing 100 mM dithiothreitol and boiled in a water bath for 5 minutes. 20-50 mg of proteins were separated on SDS-PAGE (7-10% gel) followed by transfer to a PVDF membrane. Membranes were blocked for 1 hour at room temperature in TBST 5% non-fat dry milk followed by an overnight incubation with the anti-phospho-Ser473-PKB, anti-PKB, anti-α-actin (Cell signaling) or anti-GLUT4 (Abcam). Blots were then incubated with an HRP-conjugated goat anti-rabbit secondary antibody for 1 hour at room temperature. Bands were visualized by ECL and quantified using Image J (NIH, USA).

### Statistical analysis

All data derived from the experiments were analyzed using the SPSS 15.0 package. DEXA scan data and indirect calorimetry data was tested by one-way ANOVA for normally distributed data followed by a Tukey's multiple comparison test between the HFP, HFL and HFPS groups. Indirect calorimetry data was split into day and night values for all respiratory parameters as well as activity levels. Clamp data were generated in two experiments; HFL versus HFP and HFPS versus HFP. CLAMP data was analyzed per experiment using unpaired student T-Test for normally distributed data. For graphical representation, data was normalized to HFP. In all graphs and tables, means ± SEM are given. Statistical significance threshold was set at p < 0.05.

## Results

### The effect of dietary stearate on whole body energy metabolism

To determine whether a high level of dietary stearate induces changes in whole body substrate selection or energy metabolism, three high fat diets were evaluated (table 1): a low stearate diet based on palm oil (HFP, containing 4.4% stearate) and two stearate rich diets based on lard (HFL, containing 15.0% stearate) or the palm oil diet supplemented with tristearin (HFPS, containing 13.9% stearate).

**Figure 1:**
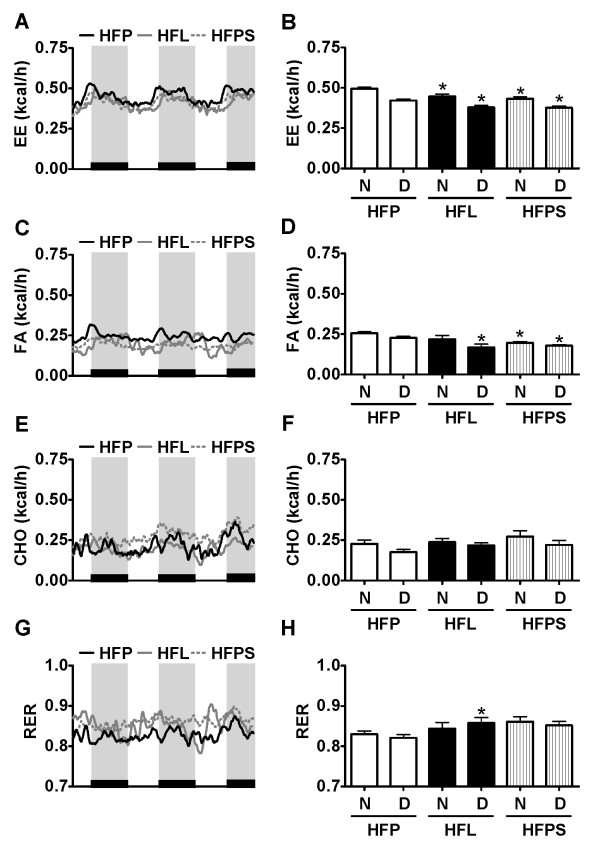
**Indirect calorimetry analyses**. Comparison of diets HFP, HFL and HFPS N = 7-8 mice per group. Rates and averaged values of energy expenditure (A, B), fatty acid oxidation (C, D), carbohydrate oxidation (E, F) and Respiratory Exchange Rates (G, H) over a period of 2.5 days and 2.5 nights. Night periods are indicated by the black bars on the x-axis of the line graphs. In the line graphs, solid black lines represent the HFP group, solid grey lines the HFL group and the dotted grey lines the HFPS group. In the bar graphs, nocturnal versus diurnal averaged values are indicted with, respectively, N and D. * = p < 0.05 for the comparison with the equivalent period of the day of HFP fed mice.

Mice were fed the various diets for 5 weeks and subjected to indirect calorimetry using automated metabolic cages. The animals fed the stearate rich HFL and HFPS diets exhibited significant lower energy expenditure rates compared to animals fed the HFP diet (figure 1A). The lower energy expenditure levels in HFL and HFPS fed animals was associated with lower caloric energy expenditure levels during both the diurnal and nocturnal period of the day in HFL and HFPS fed animals (figure 1B). Activity levels did not differ between groups at any part of the day (data not shown). These data indicate that the lower energy expenditure levels were independent of physical activity. The significant decrease in accumulated energy expenditure was mainly due to a significantly lower FA oxidation rate (figure 1C, D). Nocturnal FA oxidation rate tended to be lower in HFL fed animals compared to HFP fed animals, although this failed to reach statistical significance (p = 0.08). Carbohydrate oxidation was not different between groups, at any time of the day (figure 1E, F). During the diurnal period, RER values only differed significantly between the HFL group and the HFP group, whereas nocturnal values did not differ between groups (figure 1G, H). This may be due to the large variation in absolute carbohydrate oxidation compared to absolute fat oxidation within the HFP and HFPS groups. Since the RER represents a composite measure of carbohydrate and fat oxidation, the variation in carbohydrate oxidation will also affect variation in RER.

Accumulated food intake over a period of 2.5 days was significantly higher in HFL fed animals but not HFPS fed animals compared to HFP fed animals (HFL, 8.4 ± 0.7 g; HFPS, 8.3 ± 0.7 g and HFP 7.2 ± 0.8 g, respectively, p = 0.02 for HFL versus HFP). These data show that mice fed the stearate rich HFL and HFPS diets have higher or equal caloric intake but lower caloric expenditure compared to the low stearate HFP fed mice, a metabolic state favoring fat deposition. Accumulated fecal caloric content determined in HFP and HFPS fed animals after the end of the metabolic cage experiment did not differ significantly between groups (HFP, 7.0 ± 1.9 kcal, HFPS, 7.8 ± 2.8 kcal, p = 0.520). After 5 weeks of high fat diet, total body mass was higher in HFL and HFPS fed mice when compared to HFP fed mice (HFL, 37.0 ± 3.0 g, HFPS, 35.6 ± 2.9 g, HFP, 31.1 ± 2.3 g resp., p < 0.05). DEXA analysis revealed that this was due to a significantly higher fat mass in HFL and HFPS fed mice (HFL, 15.3 ± 2.8 g, HFPS, 14.3 ± 1.2 g, HFP, 9.9 ± 1.4 g resp., p < 0.05). Lean body mass did not differ significantly between groups (data not shown). In addition, bone mineral content and bone mineral density did not differ between groups (data not shown). Taken together, these data show that the metabolic state of mice fed the stearate rich HFL and HFPS diets resulted in higher adiposity when compared to low stearate HFP fed mice.

### The effect of dietary stearate on insulin sensitivity

To determine whether the adverse effects of stearate on whole body metabolism are associated with the deterioration of tissue specific insulin sensitivity, hyperinsulinemic euglycemic clamp analyses were performed.

**Figure 2:**
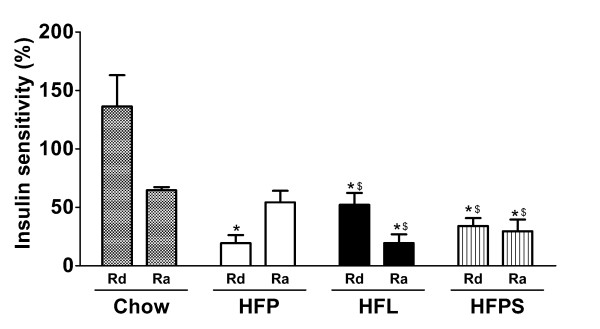
**Insulin sensitivity**. Chow, HFP, HFL and HFPS fed mice were subjected to hyperinsulinemic-euglycemic clamp analysis. Shown are the insulin sensitivity of the peripheral tissues and the liver. Peripheral insulin sensitivity was expressed as the percentage of increase of glucose disappearance rate (Rd) during the hyperinsulinemic state compared to basal. Hepatic insulin sensitivity was expressed as the percentage of repression of hepatic glucose production compared to basal. N = 5-8 per group. The chow data (checkered bars) were obtained in a separate experiment and are shown for comparison. *; p < 0.05 compared to chow, ^$^; p < 0.05 compared to HFP.

Fasting plasma glucose levels did not differ significantly between groups (HFL; 4.4 ± 0.4, HFP, 4.7 ± 0.7 and HFPS 4.0 ± 0.4 mmol/l). At the end of the hyperinsulinemic period, plasma glucose levels where somewhat higher in the HFL group, but did not differ significantly from HFP controls (HFL; 5.3 ± 0.5, HFP, 4.4 ± 0.8 and HFPS 4.2 ± 0.3 mmol/l). Insulin levels did not differ between the HFL and HFP groups at the start (HFL; 0.6 ± 0.2, HFP, 0.8 ± 0.3 ng/mL) or at the end of the clamp (HFL; 4.7 ± 1.7, HFP, 4.4 ± 0.8 ng/mL). Unfortunately, due to experimental error, insulin levels could not be determined in the HFP versus HFPS experiment. During the hyperinsulinemic clamp, the steady-state glucose infusion rate was significantly decreased in HFP, HFL and HFPS fed mice compared to a historical reference group of chow fed mice (Chow; 124.6 ± 25.4, HFP; 34.5 ± 22.6, HFL; 35.1 ± 17.4 and HFPS; 46.5 ± 22.6 umol*min^-1^*kg^-1^, p < 0.05 for all groups compared to chow), demonstrating that all high fat diets induce whole body insulin resistance (supplemental figure 1b). Interestingly, the ability of insulin to repress hepatic glucose production was significantly decreased in HFL and HFPS fed animals compared to HFP fed mice (figure 2). Insulin mediated uptake of glucose by peripheral tissues was lower in all high fat diet groups compared to chow controls (figure 2). In addition, the ability of insulin to stimulate the rate of disappearance of glucose was significantly reduced in HFP fed animals compared to HFL and HFPS fed animals, indicating aggravated insulin resistance in peripheral tissues (figure 2). These data show that despite a similar decrease in whole body insulin sensitivity, the insulin resistance induced by the low stearate HFP diet is characterized by peripheral insulin resistance only. In contrast, the stearate rich HFL and HFPS diets induce severe hepatic insulin resistance but a relatively less severe peripheral insulin resistance.

**Figure 3:**
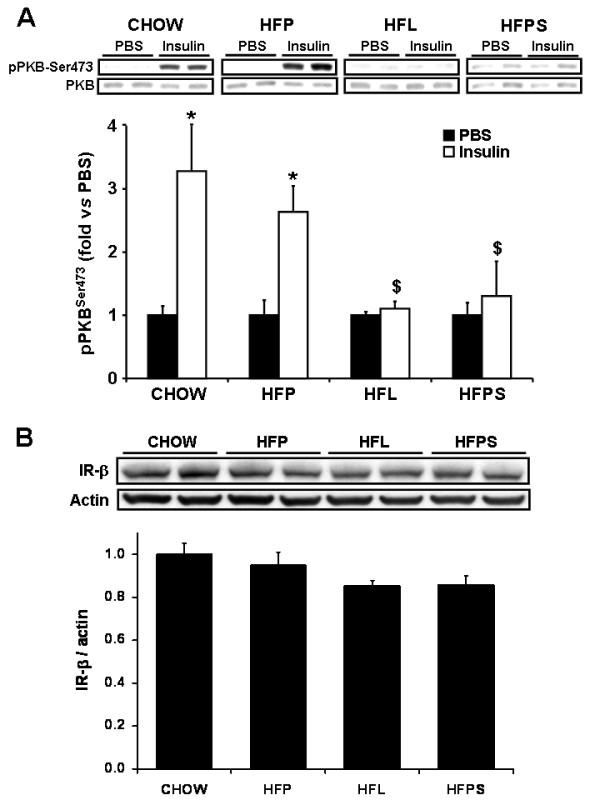
**Insulin-induced phosphorylation of PKB and IR-β expression in liver**. Chow, HFP, HFL and HFPS fed mice were subjected to continuous PBS or insulin (0.25 mU/min) i.v. infusion for 15 min before organ collection. Extracts from livers were immunoblotted with anti-phospho-Ser 473 (A) and anti-IRβ antibodies (B). Total PKB and α-actin were used as loading controls, respectively. The results of densitometric quantification are expressed in arbitrary units as the ratio over the chow group and are means ± SEM, n = 7-8. *,$ *p *< 0.05 compared with PBS and chow, respectively.

To further investigate the stearate induced alterations in tissue specific insulin sensitivity, insulin signaling was examined in liver and skeletal muscle (calf muscle) from mice subjected to a 15 min i.v. infusion of PBS (control) or insulin. In liver, the insulin-induced stimulation of PKB^ser473 ^phosphorylation was identical in chow and low stearate HFP fed mice (3.3 ± 0.6 and 2.9 ± 0.4 fold, respectively) but severely impaired in stearate-rich HFL and HFPS fed mice (1.1 ± 0.1 and 1.1 ± 0.4 fold, respectively; *p *< 0.01 and p < 0.05 compared to HFP fed mice) (figure 3A). This is in agreement with the clamp data on hepatic insulin sensitivity. In parallel, hepatic expression of the insulin receptor β (IR-β), which mediates cellular insulin action, tended to be decreased in livers from mice fed stearate rich HFL and HFPS, although this did not reach statistical significance (p = 0.11 and p = 0.13 in HFL and HFPS versus HFP, respectively) (figure 3B).

**Figure 4:**
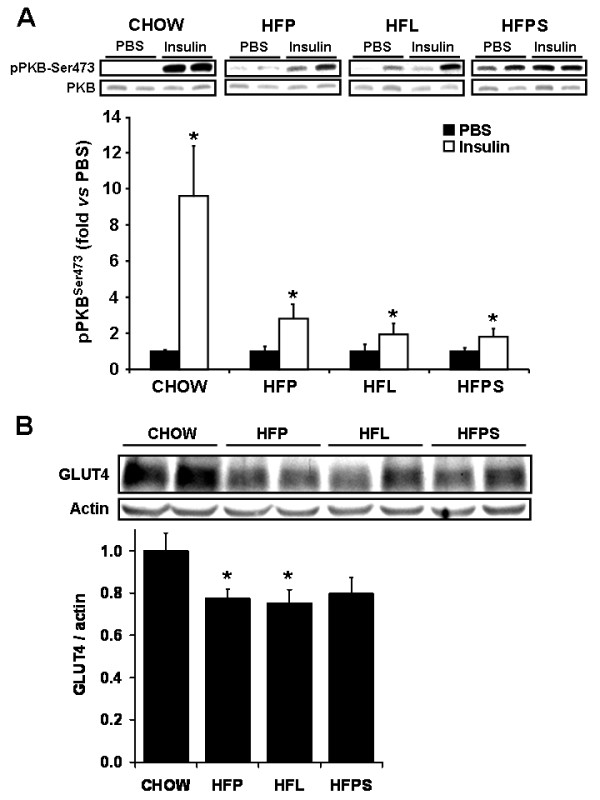
**Insulin-induced phosphorylation of PKB and IR-β and GLUT4 expression in calf muscle**. Chow, HFP, HFL and HFPS fed mice were subjected to continuous PBS or insulin (0.25 mU/min) i.v. infusion for 15 min before organ collection. Extracts from calf skeletal muscle were immunoblotted with anti-phospho-Ser 473 (A), and anti-GLUT4 antibodies (B). Total PKB and α-actin were used as loading controls, respectively. The results of densitometric quantification are expressed in arbitrary units as the ratio over the chow group and are means ± SEM, n = 7-8. *,$ *p *< 0.05 compared with PBS and chow, respectively.

In skeletal muscle, the insulin induced stimulation of PKB^ser473 ^phosphorylation was impaired to a similar extent by all high fat diets compared to chow (2.8 ± 0.8, 1.9 ± 0.6, 1.8 ± 0.4 and 9.6 ± 3.0 fold for HFP, HFL, HFPS and chow, respectively; *p *< 0.05), although no differences could be detected between the high fat groups (figure 4A). Similarly, total GLUT-4 content was reduced in the high fat diet groups HFP and HFL compared to chow (p = 0.03, p = 0.03 in HFP and HFL versus chow, respectively) and was reduced in HFPS, although this did not reach statistical significance (p = 0.06). No differences could be detected between high fat diet groups (p = 0.39 and p = 0.40 in HFL and HFPS versus HFP, respectively). These data are in line with the clamp results indicating severe impairment of peripheral tissue insulin sensitivity by the high fat diets.

## Discussion

In this study, we have addressed the role of stearate in high fat diet induced obesity and insulin resistance. As compared to the low stearate HFP diet, the HFL diet naturally high in stearate as well as the HFPS diet exogenously enriched with stearate (table 1) resulted in lower energy expenditure (figure 1). Energy expenditure values were lower during both the active (nocturnal) and inactive (diurnal) part of the day, indicating that the lower energy expenditure was independent of activity. The lower energy expenditure levels were characterized by a lower fat oxidation. Food intake was either higher (HFL) or similar (HFPS) compared to HFP fed mice. Weight gain was higher in the HFL and HFPS fed animals as compared to HFP fed animals. These results may be explained by a low oxidative efficiency of stearic acid which, together with the differences in food intake, may lead to changes in nutrient partitioning and subsequent storage of fat in white adipose tissue.

In addition to an adverse metabolic phenotype, high dietary stearate levels decreased hepatic insulin sensitivity, characterized by a decreased repression of hepatic glucose production (figure 2) and impaired induction of hepatic PKB^ser473 ^phosphorylation by insulin (figure 3A). Thus, high fat diets rich in stearate induce a metabolic state favoring adipogenesis and hepatic insulin resistance.

The low stearate HFP diet did not affect hepatic insulin sensitivity as determined by clamp analysis and quantification of insulin induced PKB^ser473 ^phosphorylation (figure 3, 4A). Addition of stearate to HFP in the HFPS diet mimicked the effects of the high stearate HFL diet on hepatic insulin sensitivity. Thus, stearate enrichment of the HFP diet per se induced an adverse metabolic phenotype and hepatic insulin resistance. However, since the FA composition of HFPS and HFL are not identical (table 1), we cannot exclude that other FA's or non fatty acid components of the HFL diet, in addition to stearate, contributed to the induction of hepatic insulin resistance in HFL fed animals. However, although food intake was significantly higher in HFL fed animals compared to HFP fed animals, no differences were found between HFPS and HFP fed animals. This indicates that the amount of stearate added to the diet does not affect food intake, and thus, that the hepatic insulin resistance found in the HFPS fed animals is dependant of dietary fatty acid composition rather than food intake.

Interestingly, the effects of high dietary stearate are in line with the effects of deficiency for Elovl6, the gene encoding the elongase that catalyzes the conversion of palmitate to stearate [19]. Deficiency for *Elovl6 *protected high fat diet fed mice from hyperinsulinemia and hyperglycemia but not obesity and steatosis. These data underline that fatty acid composition rather than fat content per se determines insulin sensitivity in liver.

All high fat diets decreased whole body insulin sensitivity (figure 2). This was characterized by a significantly reduced insulin stimulated uptake of glucose in peripheral tissues. Interestingly, insulin sensitivity of peripheral tissues was significantly more affected in mice fed the low stearate diet as compared to the two stearate rich diets. Analysis of insulin induced phosphorylation of PKB^ser473 ^in muscle confirmed that all high fat diets induce peripheral insulin resistance (figure 4B). However, this analysis did not confirm a difference in peripheral insulin sensitivity between the low stearate and stearate rich diets. This discrepancy between the clamp and the PKB^ser473 ^phosphorylation analyses in muscle could be due to the differences in the sensitivity of each method (whole body/steady state versus isolated tissue/bolus injection). In addition, it is possible that the glucose and insulin response of PKB^ser473 ^phosphorylation follow different kinetics in response to a continuous infusion and a bolus administration. Moreover, alternative mechanisms responsible for the induction of insulin resistance could play a role in peripheral tissues.

The mechanistic explanation for the induction of hepatic insulin resistance by dietary stearate remains to be determined. The higher food intake in the HFL fed animals *per sé *may induce insulin resistance. However, no differences in food intake were found between the HFP and HFPS fed groups. As the only difference in dietary composition between these groups is stearate, these data indicate that the higher food intake in the HFL fed group are due to other components than stearate. In addition, these data indicate that stearate induces hepatic insulin resistance independent of food intake. In a number of studies, long chain saturated fatty acids have been shown to induce insulin resistance by activation of pathways involved in inflammation that intersect with insulin resistance such as the Toll-like receptor 4 mediated activation of NF-kB, as well as hyperphosphorylation of protein kinases like mammalian target of rapamycin (mTOR), c-jun N-terminal kinase (JNK), and protein kinase C isoforms [20-22]. A sustained activation of these signaling kinases has been linked to abrogation of the activation of the PI3K-PKB/Akt pathway by insulin by inducing inhibitory serine phosphorylations on the insulin receptor and IRS1/2 [23]. The involvement of these pathways in the development of insulin resistance in the various tissues is currently under investigation.

A second explanation could be that stearate has been found to be poorly oxidized in isolated rat hepatocytes compared to myristate (C14:0) and palmitate (C16:0) [24]. Since stearate is also a poor substrate for the generation of triglycerides and subsequently VLDL synthesis [25], this could lead to an increased level of hepatic stearate and/or stearate derived intermediates such as diacylglycerol [26]. Accumulation of these factors has been linked to increased insulin resistance (reviewed in [27]).

A third explanation for the stearate effect concerns the role of saturated fatty acids in determining membrane rigidity and fluidity. The FA saturation degree and FA chain length as well as the relative abundance of individual FA have been described to affect membrane composition and rigidity/fluidity [28]. This is especially true in tissues where FA represent a large proportion of the membrane, such as the liver in which FA can make up as much as 10% of the total membrane [29]. *In vitro *modeling studies of artificial cholesterol/phospholipid membranes reveal that, already at a low concentration, stearate destabilizes membrane integrity by increasing the rigidity [30]. The stearate rich diets HFL and HFPS could thus affect hepatic membrane structure, which in turn will affect insulin signal transduction across the plasma membrane.

In conclusion, our findings clearly show that feeding high fat diets rich in stearate for 5 weeks induces hepatic insulin resistance and obesity. Unraveling the mechanisms underlying the effect of stearate on the liver and on the development of obesity is highly relevant to the human, in particular given the high proportion of stearate in the human diet.

## Abbreviations

CLAMP: Hyperinsulinemic - Euglycemic Clamp Analysis; DEXA: Dual Energy X-ray Absorptiometry; FA: Fatty acids; HFL: High Fat Lard diet; HFP: High Fat Palm oil diet; HFPS: High Fat Palm oil diet supplemented with Tristearin; HGP: Hepatic Glucose Production; LFL: Low Fat Lard diet; LFP: Low Fat Palm oil diet; VCO2: Carbon dioxide production rate (ml/hr); VO2: Oxygen consumption rate (ml/hr); RER: Respiratory Exchange Ratio; TLR4: Toll Like Receptor 4

## Competing interests

The authors declare that they have no competing interests.

## Authors' contributions

SVDB wrote the paper, designed the research, conducted the research and analyzed the data. BG wrote the paper, conducted the research and analyzed the data. SB conducted the research. DMO conducted the research. PJV conducted the research and analyzed the data. RRF provided essential reagents and wrote the paper. LMH provided essential reagents and wrote the paper. JAR provided essential reagents and wrote the paper. KWVD wrote the paper, designed the research and had primary responsibility for final content.

All authors have read and approved the final manuscript

## Supplementary Material

Additional file 1 **Time course of plasma glucose and glucose infusion rate during hyperinsulinemic euglycemic clamp**. Plasma glucose levels and glucose infusion rates as recorded during the hyperinsulinemic euglycemic clamp analysis. Click here for file
