# Supplementary figures and images for: High levels of dietary stearate promote adiposity and deteriorate hepatic insulin sensitivity
Source: Nutr Metab (Lond). 2010 Mar 27;7:24. doi: 10.1186/1743-7075-7-24 (PMC2852377; doi:10.1186/1743-7075-7-24)

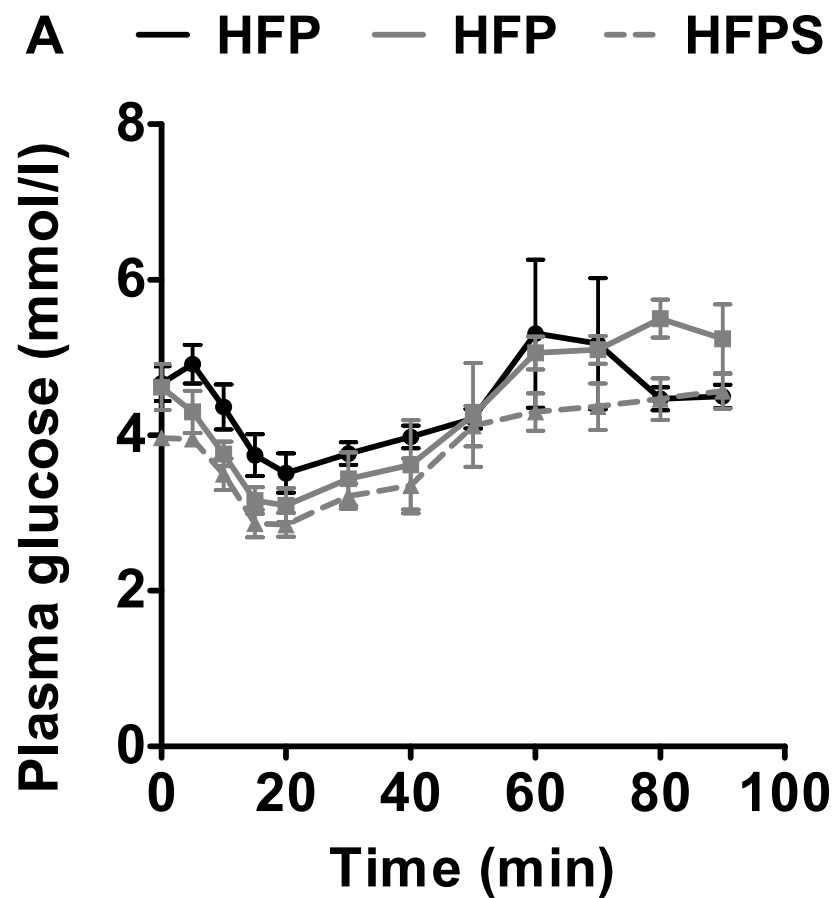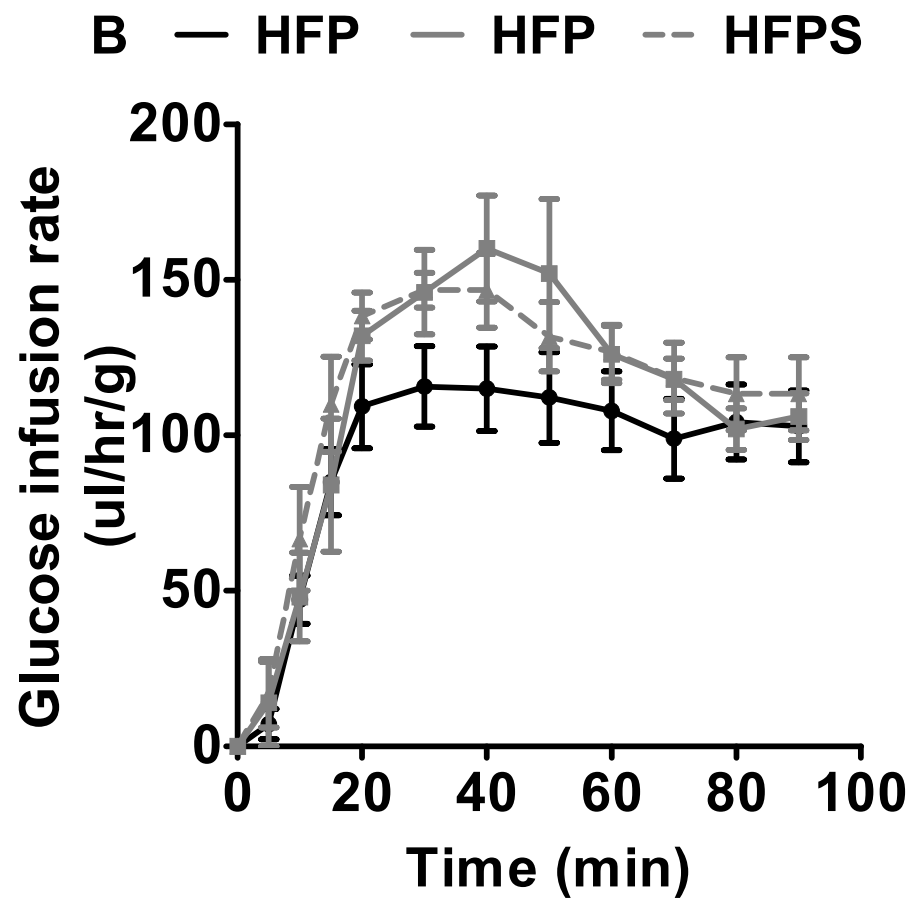

Supplement: Additional file 1 — Time course of plasma glucose and glucose infusion rate during hyperinsulinemic euglycemic clamp. Plasma glucose levels and glucose infusion rates as recorded during the hyperinsulinemic euglycemic clamp analysis. [file 1743-7075-7-24-S1.PDF]
